# Supplementary material for: A multi-omics longitudinal aging dataset in primary human fibroblasts with mitochondrial perturbations
Source: Sci Data. 2022 Dec 3;9:751. doi: 10.1038/s41597-022-01852-y (PMC9719499; doi:10.1038/s41597-022-01852-y)
Supplement: Supplementary file 1 — Supplemental File 1 [file 41597_2022_1852_MOESM1_ESM.pdf]

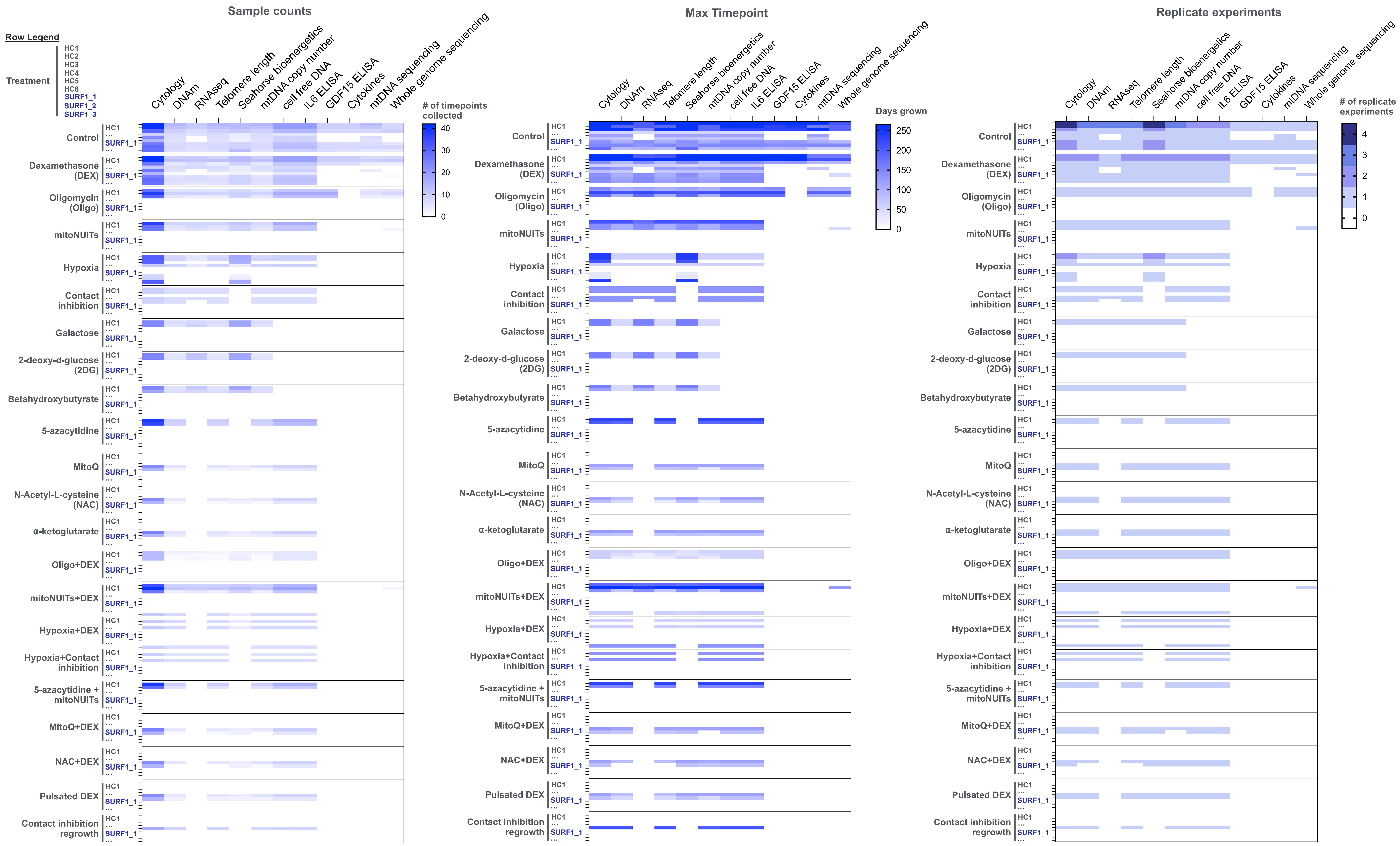

Supplemental Figure 1. Sample counts, max timepoint, and replicated experiment counts for each measured parameter. (**A**) Heatmap of the number of timepoints for each parameter (columns) associated with a given donor cell line and treatment (rows). (**B**) Heatmap of the maximum timepoint collected for each parameter (columns) associated with a given donor cell line and treatment (rows). The highest value is 265 days. The maximal duration of each experiment was determined by either the absence of divisions for a 30-day period, reaching the target endpoint for a specific experiment (e.g., Treatment 5: Hypoxia), or rare events of bacterial contamination. (**C**) Heatmap of the number of repeat experiments (i.e. Study Parts) for each parameter (columns) associated with a given donor cell line and treatment (rows).
